# Feasibility of monitoring stress using skin conduction measurements during intubation of newborns

**DOI:** 10.1007/s00431-015-2621-6

**Published:** 2015-09-02

**Authors:** Robin van der Lee, Liesbeth JM Groot Jebbink, Thea HM van Herpen, Esther J d’Haens, Josette Bierhuizen, Richard A van Lingen

**Affiliations:** Princess Amalia Department of Paediatrics, Department of Neonatology, Isala Clinics, Dokter van Heesweg 2, 8025 AB Zwolle, The Netherlands; Hospital Pharmacy, Isala Clinics, Zwolle, The Netherlands; Department of Neonatology, Emma Children’s Hospital Academic Medical Center, Amsterdam, The Netherlands; Department of Neonatology, Emma Children’s Hospital Academic Medical Center, Meibergdreef 15, 1105AZ Amsterdam, The Netherlands

**Keywords:** Galvanic skin response, Deep sedation, Neuromuscular blockade

## Abstract

The objective of this study was to assess the feasibility of monitoring stress responses in newborns during naso-tracheal intubation after two different premedication regimens, using skin conductance measurements (SCM). Twenty-two newborns were randomised and premedicated with morphine + vecuronium or propofol. SCM (peaks/s) were collected prior to, during and after the procedure. Threshold for interpreting responses as stressful was 0.21 peaks/s. Intubation conditions and physiological parameters were registered. Intubation conditions were good in all newborns. Administration of morphine (range 1.4–10.3 min) before administration of vecuronium did not affect SCM when a stressful stimulus was applied. Within 1.6 min (range 0.8–3 min) after administration of vecuronium, SCM disappeared in 10 of 11 newborns. Propofol reduced SCM in 10 of 11 newborns at the first attempt. Further attempts were associated with increasing SCM, mostly above a threshold of 0.21 peaks/s. There were no significant changes in physiological parameters during the procedure for either premedication regimen.

*Conclusion*: The variation in SCM between individual newborns limits the usefulness of SCM as stress monitor during intubation. The use of neuromuscular blockers for premedication precludes monitoring of SCM completely in newborns.

**Table:** 

**What is Known:** • *Skin conductance measurements have been used successfully to monitor pain in awake newborn infants.*
**What is New:** • *Premedicated newborns display significant interindividual variation in skin conductance measurements during an intubation procedure.* • *Neuromuscular blockade causes skin conductance measurements to disappear completely*.

## Introduction

A considerable number of papers has been published on a variety of premedication drugs used in term and preterm newborns before intubation. The focus of research is usually on the clinical effectiveness of these medications [15, 19].

Assessment of pain and stress in newborns is a subject of ongoing investigation since a gold standard does not exist. Several different approaches to quantifying pain in newborns have been developed. These methods rely predominantly on observational techniques and physiological parameters [6, 12].

Skin conductance measurements (SCM) seem to correlate well with observational pain assessment, which has been validated in term and preterm infants [8, 13, 20]. The sympathetic nervous system controls the palmar and plantar sweat glands by secreting acetylcholine in the postganglionic synapses. When activated SCM increase temporarily because of pulsatile sweat excretion, SCM closely follow (with a consistent delay of 1–2 s) the pattern seen during microneurographic recording of sympathetic nerve activation [2, 17]. An added advantage of this method is that stress assessment is possible in deeply sedated and, reportedly, paralysed (adult) patients [16].

Currently, there are no reports on the effectiveness of premedication drugs on pain and stress reduction in sedated and/or paralysed newborns during painful procedures.

Premedication drugs commonly used prior to intubation of term and preterm newborns include morphine, fentanyl, remifentanil, thiopental, propofol and neuromuscular blockers. The two most frequently used premedication drugs are (1) opioids in combination with a neuromuscular blocker and (2) propofol. There is increasing awareness that morphine might not be very effective in providing analgesia in an emergency setting [4, 19]. Following the publication of a small RCT, propofol is increasingly used in Dutch NICUs because of its hypnotic activity [11]. However, there are concerns regarding its adverse effects, i.e. its potential for decreasing blood pressure and thus possibly threatening cerebral perfusion [29]. Furthermore, propofol lacks analgesic activity and might not be sufficient as a single premedication drug [25].

In this feasibility study, we investigated the usefulness of SCM as surrogate marker for stress after premedication with either morphine and vecuronium or propofol during semi-elective intubation of term and preterm newborns.

## Methods

Newborns requiring semi-elective intubation were prospectively included to receive either morphine + vecuronium or propofol as premedication before naso-tracheal intubation. The reason for using these two different premedication regimens in this setting was that these drugs represent different mechanisms of action for inhibiting stress responses. Randomisation was part of the inclusion procedure for a psychological reason: to prevent group differences regarding medication use by preference of the attending physician. We used a computer-generated randomisation list. An independent research nurse placed group and number in sealed envelopes and numbered these. Upon inclusion, the next numbered envelope was opened.

Inclusion criterion was any newborn developing the need for semi-elective intubation. Exclusion criteria were hypotension or obstructive cardiac outflow tract disorders, suspicion of a metabolic disorder (all propofol-related criteria) or suspicion of a genetic syndrome (attempt at excluding newborns with possible genetic differences in autonomic nervous functioning, i.e. trisomy 21) and the presence of discomfort or pain (presence of a urinary catheter, thorax drain, etc.) or administration of analgetics (opioids, paracetamol) in the preceding 24 h. The research protocol was reviewed and approved by both our institutional medical ethics committees and, since propofol is not registered for use in this age group, also by the Dutch national medical ethics committee.

Informed consent was obtained after the procedure because of the emergent aspect of intubation. In research in emergency settings, retrospective informed consent is fully acceptable in most European countries provided specific criteria are fulfilled [5, 14, 22].

Patient characteristics, gestational age (GA), postnatal age (PNA), birth weight (BW) and intubation indication, were registered (Table 1).

**Table 1:** Patient characteristics and procedural parameters (mean ± SD)

Parameter	Morphine/vecuronium	Propofol
Gestational age (weeks)	28 ^5^/_7_ ± 2 ^5^/_7_	31 ^4^/_7_ ± 3 ^2^/_7_
Birth weight (g)	1239 ± 528	1710 ± 519
Postnatal age (days)	1.1 ± 1.9	0.3 ± 0.5
Indication for intubation: IRDS	8	11
Sepsis	2	
Exhaustion	1	
Time admin. morphine to T = 0 (min)	6.2 ± 3.2	
Time T = 0 to start intubation procedure (min)	0.8 ± 0.5	0.6 ± 0.2
Intubating condition score (max 6)	5.4 ± 0.8	4.9 ± 0.9
Baseline SCM (peaks/s)	0.16 ± 0.02	0.22 ± 0.03

### Medication

Morphine and vecuronium were both given as a bolus intravenous injection of 0.1 mg/kg. Propofol 1 % (Lipuro®) was diluted 1:1 with glucose 5 % in order to diminish pain at the injection site and then slowly administered intravenously in a dose of 2 mg/kg. If necessary, this dose could be repeated once. The dosages of all used medications are according to medication guidelines in Dutch NICUs.

### Study protocol

SCM (Med-Storm Pain Monitor, Med-storm Innovation AS, Oslo, Norway) were started as soon as the decision to intubate had been made. Timelines in the two groups studied were as follows:

- Morphine-vecuronium: Baseline SCM recording for at least 60 s; T = −10 min: morphine administration; T = 0: vecuronium administration. As soon as the intubating clinician deemed possible, the naso-tracheal intubation procedure was started. Intubating conditions were scored using a modified intubating score [10] (Table 2).

**Table 2:** Intubating condition score (ICS)

	Clinically acceptable		Not clinically acceptable
	Excellent (2)	Good (1)	Bad (0)
Reaction to introduction tube	No	Slight	Vigorous/sustained
Laryngoscopy	Easy	Fair	Difficult
Position of vocal cords	Abducted	Intermediate/moving	Closed

Adapted from Fuchs-Buder et al. (2007). Acceptable: ICS 3 or higher
- Propofol: Baseline SCM recording for at least 60 s; T = 0: propofol administration. As soon as the intubating conditions were acceptable, the naso-tracheal intubation procedure was started. Intubations were performed by nurse practioners, residents or neonatologists, reflecting the setting of a major teaching hospital.

### Skin conductance measurements (SCM)

The following specific events were recorded: the moment the medication was administered, any painful or stressful stimuli (removal of adhesive plaster, suction, non-invasive ventilation, insertion of tube and laryngoscope, and the moment intubation was completed successfully. Measurements were recorded continuously up to 30 min after completion of the intubation procedure. The parameter used for assessing SCM was the number of peaks/s, which is the best validated skin conductance parameter for pain score in newborns. This parameter represents the number of peaks within a time window and corresponds to the number of bursts per second in a skin sympathetic nerve [2, 17]. When correlated with Prechtl’s general movement observations, a cut-off point of 0.21 peaks/s reflects the threshold at which stress may become unacceptable [24]. We recorded the number of peaks/s in consecutive time windows of 15 s. A group size of ten patients in each group was deemed sufficient for assessment of feasibility of SCM during intubation.

### Physiological parameters

Heart rate, pulse oximetry and arterial blood pressure (in case an arterial indwelling catheter was present) were continuously recorded on a central monitoring system up to 30 min after completion of the intubation procedure. When no indwelling arterial catheter was present, blood pressure was measured non-invasively every 5 min.

### Data analysis

All data were analysed using the software program GraphPad Prism 5.01 (GraphPad Software Inc., San Diego, CA, USA). The differences in the number of peaks/s at the various time points of the procedure during each procedure were analysed statistically, using the non-parametric one-way ANOVA test; Dunn’s multiple comparison test was performed in order to find statistical differences between each of the analysed time points.

## Results

**Fig. 1:**
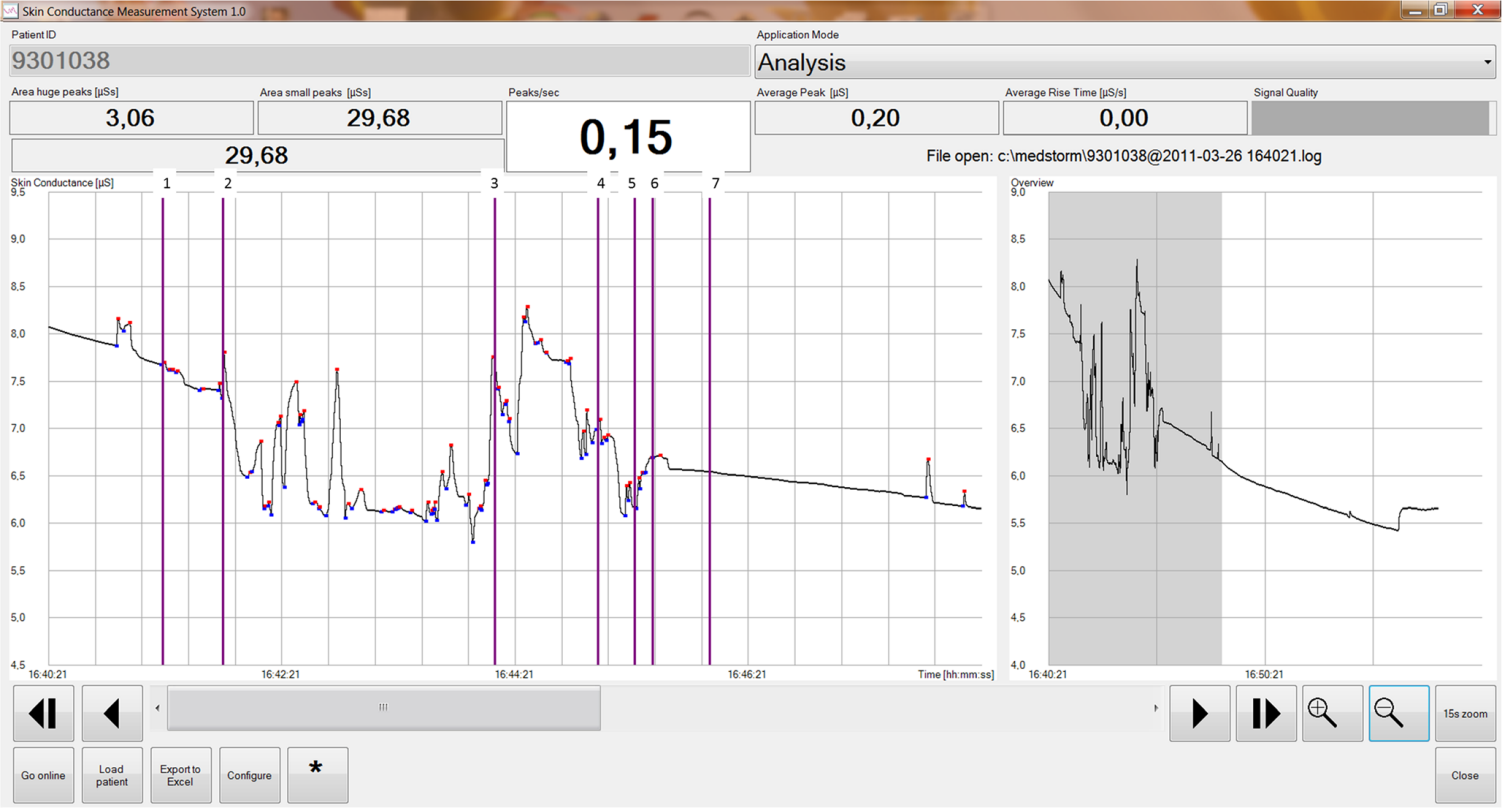
Graphical presentation of skin conduction (mean peaks/s ± SD) in relation to events in all patients in the morphine/vecuronium group. *Each colour* represents the number of patients (*between brackets*) intubated at the first, second or third attempt, respectively. The high SD values reflect substantial interindividual variation

**Fig. 2:**
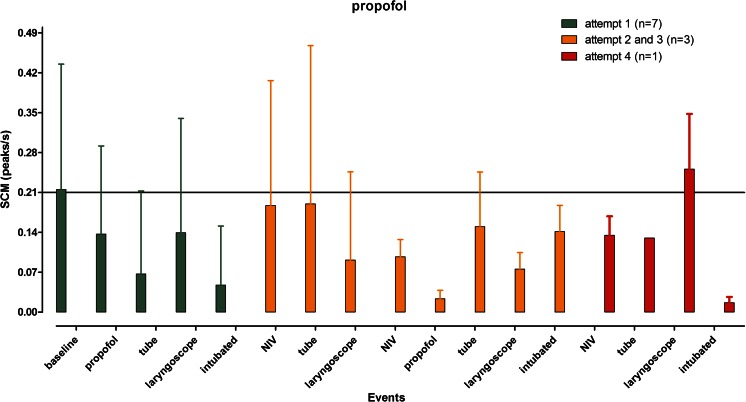
Graphical presentation of skin conduction (mean peaks/s ± SD) in relation to events in all patients in the propofol group. *Each colour* represents the number of patients (*between brackets*) intubated at the first, third (no second attempt was successful) or four attempt, respectively. The high SD values reflect substantial interindividual variation

**Fig. 3:**
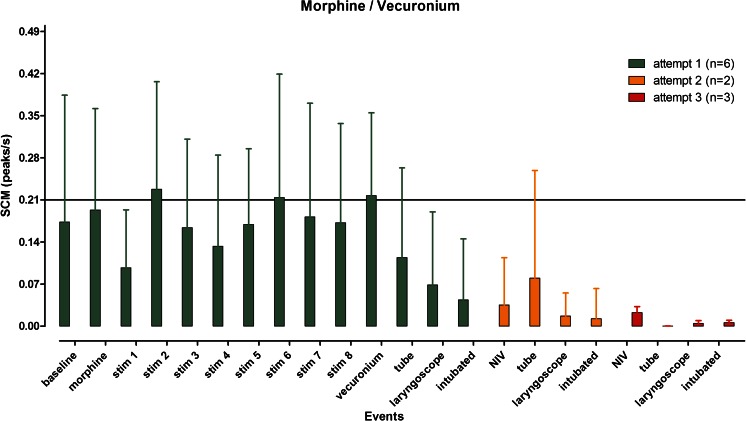
Representative recording of a patient in the MV group. The *blue and red dots* represent peaks in SCM on a baseline of conductance (μS). *Purple lines* are annotations of events: 1, morphine administration; 2, repositioning of the infant; 3, removal of adhesive tape; 4, vecuronium administration; 5, nasal insertion of the tube; 6, introduction of laryngoscope; 7, intubated

Originally, 20 newborns were to be included and randomised. However, due to technical problems with data storage on the SCM apparatus, the randomisation period was extended after conferring with our institutional medical ethics committee. In total, 28 newborns were included and randomised; in two cases, informed consent was denied. Of the 26 remaining newborns, 13 received morphine and vecuronium and 13 received propofol as premedication. In both groups, two recordings were lost due to technical difficulties and these patients were excluded. A total of 22 newborns (11 in each group) were analysed. Patient characteristics and procedural parameters are presented in Table 1. In each group, one patient exhibited a very high baseline and a significantly higher number of peaks/s both at rest and during painful stimuli resulting in higher standard deviations of the means without significant effect on the means. Interindividual variation was substantial as reflected by the coefficient of variation for the baseline values: 61 ± 49 % (range 0–141 %) and 50 ± 54 % (range 0–200 %).Also intraindividual variation was present as reflected in Fig. 1: not all increase in peaks in SCM can be explained by the applied stimuli. Morphine was administered 6.2 ± 2.7 min (mean ± SD; range 1.4–10.3 min) before vecuronium instead of at least 10 min before vecuronium as described in our protocol (in nine patients, vecuronium was administered earlier than 10 min after morphine administration). Clinical deterioration and the emergent nature of the procedure were given as the reasons for this breach of protocol. Administration of morphine before administration of vecuronium did not have any effect on SCM when a stressful stimulus (not related to intubation; for instance, removal of adhesive tape) was applied (median 0.18 peaks/s; range 0.00–0.80 peaks/s). Within 1.6 min (range 0.8–3 min) after administration of vecuronium, SCM disappeared in 10 of 11 newborns (median 0.00 peaks/s; range 0.00–0.27 peaks/s). This precluded further analysis of the effect of morphine on SCM during the intubation procedure (Fig. 2). Propofol was effective in reducing SCM in 10 of 11 newborns at the first attempt (median 0.04 peaks/s; range 0.00–0.33 peaks/s). Further attempts were associated with increasing SCM, mostly above a threshold of 0.21 peaks/s which were not influenced by a second dose of propofol (Fig. 3). Intubating condition scores remained clinically acceptable throughout the procedure, but for one patient in the propofol group (the only patient intubated after the fourth attempt). Analysis of blood pressure, heart rate and oxygen saturation revealed no significant differences from baseline measurements (data not shown). One patient receiving propofol required a fluid bolus because of arterial hypotension.

## Discussion

The two main findings in this study were as follows:

1. Significant variation in SCM both between individual as within individual patients is present in comparable conditions.
2. Neuromuscular blockade with vecuronium reduces SCM to zero within approximately 3 min.

We observed temporal relations between possible stressful stimuli and SCM in 20 out of 22 analysed patients. This temporal relationship has been reported by other authors [8, 16, 20]. However, in each group, one patient displayed a significantly higher baseline activity that was only minimally reduced by premedication regimens or increased by stimuli. Interestingly, these patients reacted clinically similar to the other patients. The expected interindividual variability in sympathetic tone is apparently reflected in a significant variance in skin conductivity. Intraindividual variation was also present as depicted in Fig. 1: Peaks in SCM appear not only after (potential) stressful stimuli but also spontaneously. Both interindividual and intraindividual variation of SCM might make it difficult to draw conclusions from the measurements in clinical situations.

Morphine is an analgesic opiate with mild sedative effect when given continuously [1]. In the acute setting, the effectiveness is questionable, most likely due to its relatively slow onset of action. This might explain that responsiveness to stressful stimuli before intubation still resulted in increased SCM. After vecuronium administration, the SCM all but disappeared within 3 min, precluding further analysis of the effects of morphine on SCM. The unexpected decline in SCM after administration of vecuronium is very difficult to explain. Although morphine might play a role in the disappearance of SCM after administration of vecuronium, this is not likely, both because of its slow onset of action and the universal disappearance of SCM regardless of the time interval between morphine and vecuronium administration. In adult patients, no disappearance of SCM is observed during intubation with sedatives and rocuronium [16]. Therefore, immaturity of presynaptic or postsynaptic receptors involved in palmar sudomotor activation is most likely the reason for the observed effect. Unfortunately, although maturation has been described in the literature for various receptors, including the postsynaptic nicotinic Ach-R, no specific information is available on maturation of the sudomotor response [9]. Muscarinic (M3) receptors are involved in the activation of the sudomotor response and pathways for noradrenergic involvement have been documented [27, 28]. In 1994, Norel et al. showed that vecuronium could inhibit M3 receptors in human airways [18]. However, the absence of a measurable effect of neuromuscular blockade on SCM in adult patients seems to indicate that direct M3 inhibition at the sudomotor junction cannot be held accountable for the observed effect in premature babies. To complicate matters, SCM involves not just the sudomotor response but also the sympathetic vasomotor response [7]. In much earlier publications, galvanic skin responses could still be recorded in patients with congenital absence of sweat glands and even post mortem, obscuring the picture of the physiology behind SCM even further [21, 23, 26]. In summary, there is a robust relation between SCM and sympathetic nerve activity but which of the several mechanisms involved in SCM is affected by administration of vecuronium in our premature babies remains unclear.

Propofol inhibits the sympathetic nervous system centrally and causes deep hypnosis (primarily, but not exclusively, via the gamma-aminobutyric acid (GABA_A_) system) but has no known analgesic effect. In newborns, the neuronal chloride gradient which is critical to GABA_A_ function is still immature: stimulation of GABA_A_ receptors then causes excitation instead of the inhibitory response seen in the mature condition. Maturation of GABA_A_ receptors (reversal of the chloride gradient) occurs in a caudo-rostral direction. It is known that in neonatal convulsions, treatment with benzodiazepines usually leads to cessation of clinically apparent convulsions while an EEG can still show epileptic activity [3]. Analogous to this observation, dissociation between peripheral and central GABA_A_ responsiveness might well explain our observations: initially adequate sedation with good muscle relaxation. However, as the procedure continued muscle relaxation persisted (more mature GABA_A_), accounting for clinically acceptable intubation conditions. At the same time, central responsiveness decreased or reverted to a more excitatory state which might explain increasing SCM.

This study shows several weaknesses. This study was clearly underpowered for a thorough analysis of the unexpected findings. Specifically, the relationship between maturation (and/or postnatal age) and the unexpected almost complete block of SCM by vecuronium could not be analysed in depth. Although very interesting the unexpected findings did not warrant an increase of the size of the groups as these findings would not answer our original question. The lack of conforming to study protocol regarding the time between the administration of morphine and vecuronium was unfortunate but in accordance with the clinical situation of impending respiratory failure.

## Conclusions

The observed interindividual and intraindividual variation in SCM in our newborns limits the usefulness of SCM as stress monitor during intubation. The use of neuromuscular blockers for premedication precludes monitoring of SCM completely in newborns.

## Abbreviations

GABA: Gamma-aminobutyric acid
SCM: Skin conductance measurements
